# Zebrafish model for functional screening of flow-responsive genes controlling endothelial cell proliferation

**DOI:** 10.1038/s41598-024-77370-1

**Published:** 2024-12-03

**Authors:** George Bowley, Sophie Irving, Imo Hoefer, Robert Wilkinson, Gerard Pasterkamp, Hazem M. S. Darwish, Stephen White, Sheila E. Francis, Tim Chico, Emily Noel, Jovana Serbanovic-Canic, Paul C. Evans

**Affiliations:** 1 School of Medicine and Population Health, University of Sheffield, Sheffield, UK; 2 Central Diagnostic Laboratory, UMC Utrecht, Utrecht, The Netherlands; 3 Faculty of Medicine & Health Sciences, University of Nottingham, Nottingham, UK; 4 Faculty of Medical Sciences, Biosciences Institute, University of Newcastle, Newcastle upon Tyne, UK; 5 School of Biosciences, University of Sheffield, Sheffield, UK; 6 Biochemical Pharmacology,William Harvey Research Institute, Barts & The London Faculty of Medicine &Dentistry, Queen Mary University of London, Charterhouse Square, London, EC1M 6BQ UK

**Keywords:** Endothelial cells, Shear stress, WNK1, Proliferation, Cardiology, Cardiovascular biology

## Abstract

Local haemodynamics control arterial homeostasis and dysfunction by generating wall shear stress (WSS) which regulates endothelial cell (EC) physiology. Here we use a zebrafish model to identify genes that regulate EC proliferation in response to flow. Suppression of blood flow in zebrafish embryos (by targeting cardiac troponin) reduced EC proliferation in the intersegmental vessels (ISVs) compared to controls exposed to flow. The expression of candidate regulators of proliferation was analysed in EC isolated from zebrafish embryos by qRT-PCR. Genes shown to be expressed in EC were analysed for the ability to regulate proliferation in zebrafish vasculature exposed to flow or no-flow conditions using a knockdown approach. *wnk1* negatively regulated proliferation in no-flow conditions, whereas *fzd5*, *gsk3β*, *trpm7* and *bmp2a* promoted proliferation in EC exposed to flow. Immunofluorescent staining of mammalian arteries revealed that WNK1 is expressed at sites of low WSS in the murine aorta, and in EC overlying human atherosclerotic plaques. We conclude that WNK1 is expressed in EC at sites of low WSS and in diseased arteries and may influence vascular homeostasis by reducing EC proliferation.

**Supplementary Information:**

The online version contains supplementary material available at 10.1038/s41598-024-77370-1.

## Introduction

With no lysine (K) 1 (WNK1) is a serine/threonine kinase that controls multiple physiological functions including blood pressure control, renal function, vascular function and neural activity^1^. Mechanistically, WNK1 phosphorylates the downstream kinases SPAK and OSR1 which regulate SLC12 family cation-chloride co-transporters^2^. The WNK1/SPAK/OSR1 pathway regulates several processes coupled to cation-chloride exchange including osmoregulation and cell size, neuronal depolarisation, renal NaCl reabsorption and vasoconstriction^1^. Since renal NaCl reabsorption and vasoconstriction are central regulators of blood pressure, it is consistent that WNK1 mutations drive Gordon’s syndrome associated with hypertension in man^3^. Endothelial-specific deletion of *Wnk1* in mice caused embryonic lethality due to defects in vascularisation^4^, indicating that endothelial *Wnk1* is essential for angiogenesis and cardiac development. Similarly, knock down of *wnk1a* in zebrafish led to a phenotypic change in intersegmental vessels^5^. The mechanism underpinning the angiogenic mechanism of WNK1 involves VEGF-AKT-WNK1 signalling^2,5^ and potentially via Wnt^6^ and TGFβ^7^ pathways. In human endothelial cells (EC) WNK1-SPAK signalling has divergent effects of pro-angiogenic signalling^8^; WNK1-SPAK drives proliferation whereas WNK1-OSR controls cell migration.

Atherosclerosis develops preferentially at branches and bends of arteries that are exposed to disturbed blood flow which generates wall shear stress (WSS) with low magnitude and variations in direction^9^. These hemodynamic conditions promote atherosclerosis by inducing EC dysfunction and by preventing vascular repair through mechanisms that are only partially understood. EC proliferation is a double-edged sword; it is required for vascular repair, but excessive EC proliferation in arteries is associated with loss of EC alignment and increased intercellular gaps, which leads to increased accumulation of proatherogenic lipoproteins in the intima^10,11^. We previously mapped WSS in the porcine aorta and used transcriptional profiling to identify several putative regulators of EC proliferation enriched at a low WSS region^12^. Here we screen these genes for the ability to regulate EC proliferation using a zebrafish model.

Zebrafish have several properties that are advantageous for analysis of gene function including their small size, genetic tractability, high fecundity, ease of maintenance and relatively low cost, coupled to a high degree of evolutionary conservation with mammals^13^. We previously demonstrated that zebrafish embryos can be used to analyse the molecular mechanisms of EC apoptosis^12,14^, and recently we optimised real-time imaging techniques to quantify EC proliferation in zebrafish embryos^15^.

Here we demonstrate that blood flow maintains EC proliferation in intersegmental vessels (ISVs) and that the cessation of flow causes a reduction in EC proliferation. Screening of putative regulators revealed that *wnk1* is necessary for the suppression of EC proliferation under no-flow conditions, while *fzd5*, *gsk3β*, *trpm7*, and *bmp2a* were drivers of proliferation in vessels exposed to flow. We conclude that zebrafish embryos are useful for assessment of regulators of EC proliferation under flow and no flow conditions.

## Materials and methods

### Zebrafish strains

All experimental protocols in this study were approved by the Animal Welfare and Ethical Review Board at the University of Sheffield, UK and conducted under a UK Home Office licence. Methods adhered strictly to the UK Animals (Scientific Procedures) Act 1986 and were reported according to ARRIVE guidelines (https://arriveguidelines.org). Maintenance, manipulation and staging of wildtype and transgenic lines were carried out as described previously^12,14,16^. The following transgenic lines were used: *Tg fli1a:EGFP *^*y1*^^17^*, Tg gata1a:dsRed *^*sd2*^^18^, *Tg fli1a:LifeAct-mClover *^*sh467*^^16^, *Tg fli1a:nls-mCherry *^*sh550*^^19^*.*

### Manipulation of flow

To arrest heartbeat pharmacologically, embryos were treated with 0.65 mg/ml tricaine (Sigma-Aldrich) in E3 embryo medium for 30 min. They were maintained in E3 with tricaine (0.1 mg/ml) during imaging from 54 h post fertilisation (h.p.f.) until 70 h.p.f. This timeframe allowed imaging of the embryo trunk when ISVs were sufficiently developed to exhibit blood flow.

### Cell sorting and qRT-PCR

ECs were isolated by fluorescence-activated cell sorting (FACS) from dissociated *fli1a:EGFP-gata1a:dsRed* embryos as described previously^12^. RNA was extracted from sorted EC using a QIASHREDDER column (Qiagen) and RNeasy kit (Qiagen) and 1 µg of total RNA was subjected to cDNA synthesis using iScript reverse transcriptase (Bio-Rad). Resulting cDNA was used as a template for qRT-PCR using gene-specific primers (Supplementary Table 1) and SYBR-Green master mix (Bio-Rad) according to the manufacturers protocol. mRNA levels were calculated using the ddCT method using levels of *b-actin* mRNA to normalise total RNA levels.

### Gene silencing and rescue using synthetic mRNA

Morpholino antisense oligonucleotides (MOs; GeneTools, LLC) were diluted in sterile water and ~ 1 nl was injected into the yolk of a 1–4 cell stage embryo. All of the MOs used in this study have been published previously and are described with citations in Supplementary Table 2.

### Quantification of ISV blood flow

To quantify blood flow in the ISVs, a ZEISS Axiozoom V16 was used to image erythrocyte velocities *in gata1a:dsRed* zebrafish embryos at 72 hpf. Images were captured at 240 fps. For analysis, images were converted to binary using ImageJ threshold tool, then TrackMate^20^ was used to measure the velocity of the erythrocyte signal.

### Quantification of EC proliferation

ISVs were selected for analysis because they have a relatively simple anatomy that is suitable for real time imaging. Arterial and venous ISVs were analysed in the zebrafish embryo trunk. EC proliferation in ISVs was quantified from 54 to 70 hpf, a time where flow was consistently established in ISVs of wild-type fish. Zebrafish embryos *Tg(fli1a:NLS-mCherry)* were mounted in 1–2% agarose and time lapse imaging was carried out using a Zeiss LSM880 in Airyscan mode as described^15^. Proliferation events were defined as where the fluorescently labelled nucleus divides. The region of interest for monitoring ISVs was immediately dorsal to the yolk extension. The number of EC proliferation events in each embryo was expressed as a fraction of the total number of EC nuclei.

### *En face* staining of murine endothelium

Mice used in this study were wildtype male C57BL6/J Charles River strain aged 12 weeks. They were euthanized through anaesthetic overdose via IP injection of sodium pentobarbital. The expression levels of WNK1 were assessed at regions of murine aortic arch exposed to high (outer curvature) or low WSS (inner curvature) by *en face* staining as described previously^12^. Fixed aortic segments were tested by immunostaining using primary rabbit anti-WNK1 antibodies (Abcam) and AlexaFluor568-conjugated secondary antibodies (red). ECs were identified by co-staining using anti-CDH5 antibody conjugated to AlexaFluor488 (BD; green). Nuclei were identified using To-Pro-3 (Invitrogen). Visualisation of EC was performed by confocal scanning microscopy (Nikon A1). Aortas were analysed on the same day using identical laser and microscopy parameters. Fluorescence was quantified for multiple EC at high and low WSS regions and values averaged. To control for specific binding, isotype-matched IgG control antibodies (abcam) were used.

### Immunostaining of human atherosclerotic plaques

Studies using human cells and tissues were used in accordance to the standards set by the Declaration of Helsinki. Human carotid plaques obtained through endarterectomy surgery were collected as part of the Athero-Express Biobank collected at the University Medical Center, Utrecht. The study was approved by local medical ethics boards and was conducted with the subjects giving informed consent. Formalin fixed paraffin embedded were dewaxed by Xylene/Ethanol treatment, then antigen retrieval was performed using boiling citrate buffer (0.01 M) pH 6.0 prior to permeabilization (0.01% triton-X100 in PBS) and blocking (1% BSA). Primary antibodies targeting WNK1 (Abcam), rabbit IgG (staining control) or vWF (BD; EC marker) were applied prior to Alexafluor568 or Alexafluor488-conjugated secondary antibodies and visualisation by fluorescence microscopy.

### Statistical analysis

Data are expressed as the average of individual experiments with standard deviation or standard error of the mean and drawn using Prism (version 9). Data were tested for normality using the Shapiro–Wilk test and parametric tests (t-test, ANOVA with Tukey’s multiple comparisons post-hoc test) or non-parametric tests (Kruskal–Wallis test) used accordingly. *p < 0.05, **p < 0.01, ***p < *0.001,* ****p < *0.0001*, *ns* = *no significant difference.*

## Results

### Endothelial proliferation is reduced in intersegmental vessels by flow cessation

**Fig. 1:**
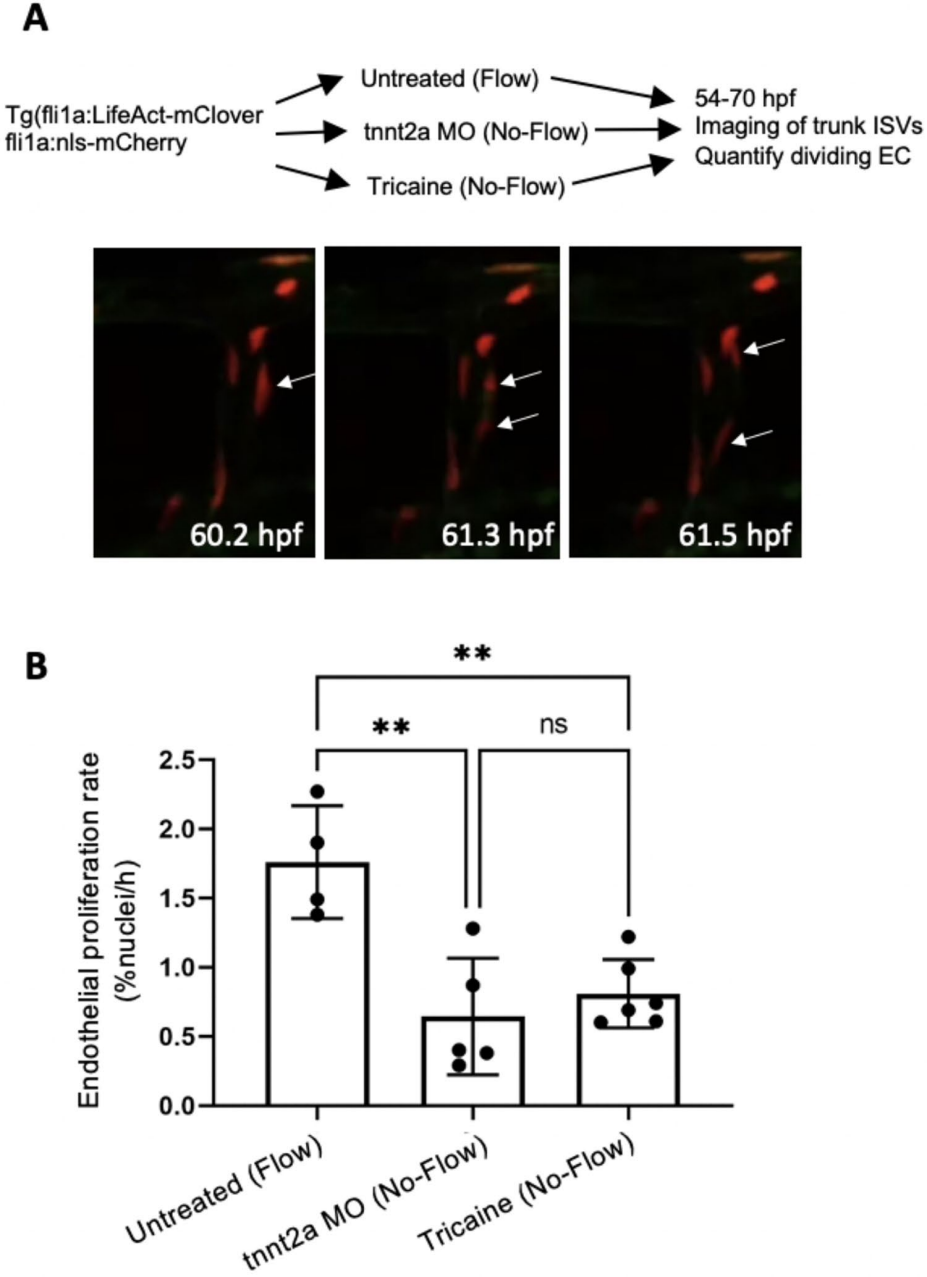
EC proliferation is significantly reduced in embryos without blood flow. Transgenic *Tg(fli1a:LifeAct-mClover; fli1a:nls-mCherry)* embryos were analysed. The ISVs of four embryos with flow, five *tnnt2a*-MO (No-Flow), and six tricaine-treated (No-Flow) embryos were imaged from 54 to 70 hpf. For each embryo, data were pooled from 7 ISVs. (**A**) Representative images showing a dividing EC (arrows) in an ISV exposed to flow. (**B**) The rate of EC proliferation in the ISVs was quantified as a percentage of nuclei dividing per hour. Mean values + /− standard deviations are shown. Differences between means were analysed using an one-way ANOVA with multiple comparisons.

The influence of WSS on EC proliferation was assessed by manipulating flow which normally commences with cardiac contraction at approximately 24 h.p.f. Blood flow was blocked using MOs targeting cardiac troponin T2 (*tnnt2a*) or tricaine to prevent heart contraction. We used this approach previously and found that embryos without cardiac contraction remain viable for several days and do not upregulate hypoxia responsive genes^21^, presumably because diffusion is sufficient for tissue oxygenation at this stage of development. It was observed by real-time confocal imaging of *Tg(fli1a:LifeAct-mClover; fli1a:nls-mCherry)* embryos that EC proliferation was significantly reduced in zebrafish lacking flow either due to tricaine treatment or as a result of *tnnt2a* knockdown (Fig. 1; Supplementary Videos 1 and 2). We conclude that suppression of flow leads to a reduction in EC proliferation in zebrafish ISVs.

### Functional screening of regulators of EC proliferation in zebrafish

**Fig. 2:**
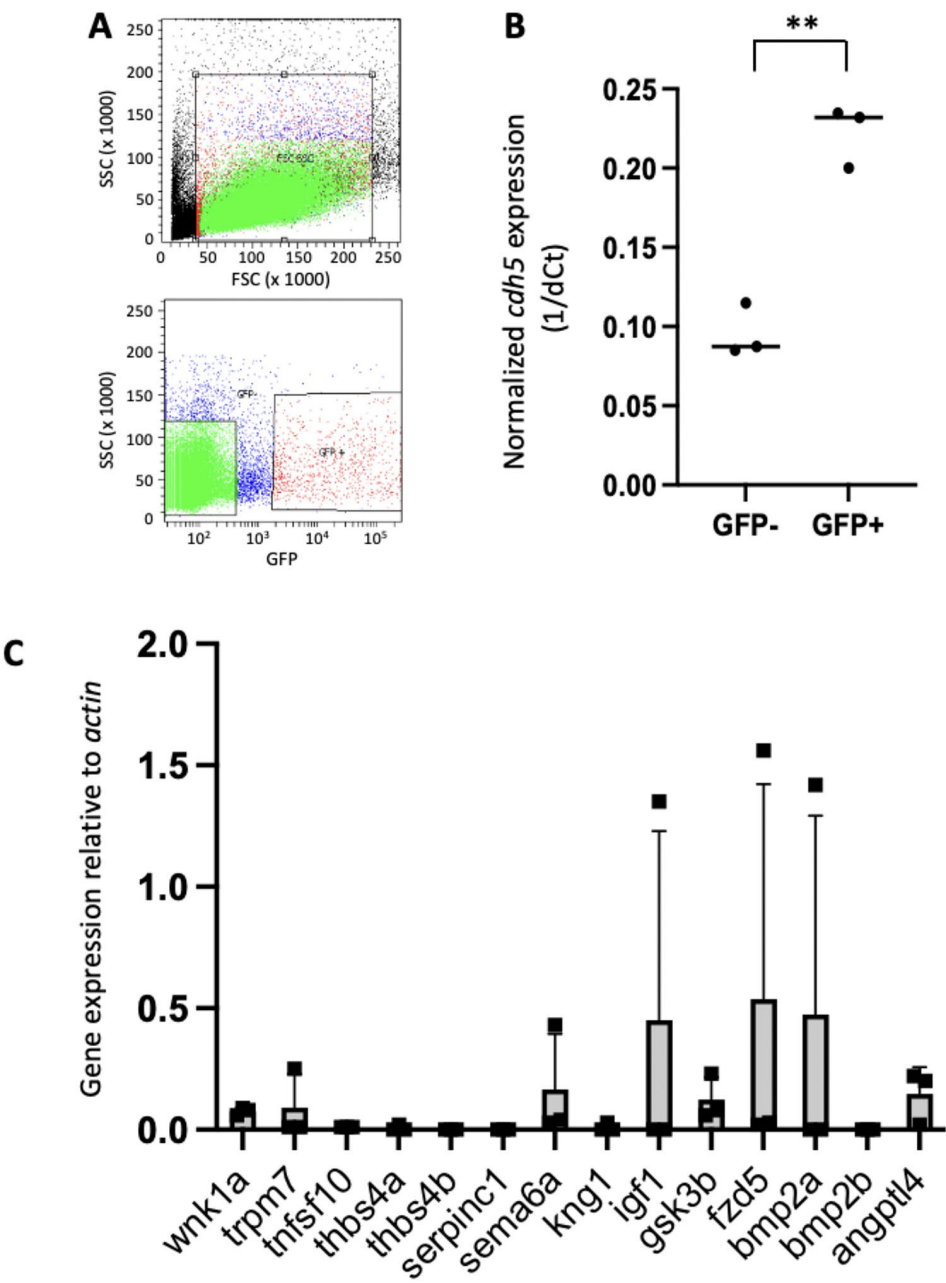
Sorting of EC from *fli1a:EGFP; gata1adsRed* zebrafish. Approximately 40 *Tg(fli1a:EGFP; gata1a:dsRed)* embryos at 48 hpf were dissociated into single cells using trypsin. Using FACS, cells were gated using forward scatter (FSC) and side scatter (SSC) and then GFP + and GFP-single cells were sorted. N = 3 independent experiments were performed. (**A**) Typical flow cytometry data showing FSC and SSC profiles (upper panel; gating indicated as red box) and delineation of GFP + and GFP- single cells (lower panel; gating indicated as red boxes). (B) *cdh5* expression was quantified in GFP + cells by qRT-PCR using *actin* as a control for total RNA levels. Mean 1/dCt values and individual data points are shown. Differences between means were analysed using a t-test. *cdh5* expression was enriched in GFP + cells. (**C**) Quantification of expression of genes of interest in zebrafish endothelium. GFP + cells were analyzed by qRT-PCR. Expression of genes of interest was quantified relative to the expression of *actin*. Mean values + /− standard deviations and individual data points are shown. Genes which were expressed at detectable levels in one or more experiment were selected for study.

Putative regulators of proliferation that were differentially expressed at low and high WSS regions of the porcine aorta^12^ were selected for functional screening based on the existence of 1–2 orthologues in zebrafish (Supplementary Table 3). To determine whether candidate genes are expressed in zebrafish EC, we collected embryos with fluorescently labelled ECs (*Tg(fli1a:EGFP;gata1a:dsRed)*), generated single cells from them, and performed FACS to isolate EC (Fig. 2A). In three experiments, FACS successfully isolated more than 900 GFP positive cells out of approximately 10^5^ total cells. It was confirmed that the sorted cells were enriched for EC by qRT-PCR analysis of *cdh5;* the levels of cdh5 were 22 times greater in in GFP-positive cells compared to the GFP-negative population (Fig. 2B). The genes *wnk1a*, *trpm7*, *sema6a*, *igf1*, *gsk3β*, *fzd5*, *bmp2a* and *angptl4* were expressed in zebrafish EC in at least one of three experiments (Fig. 2C) and therefore selected for further analysis. By contrast, *tnfsf10, thbs4a, serpinc1, kng1* and *bmp2b* were not detected in three independent experiments and were therefore excluded from further analysis (Fig. 2C).

**Fig. 3:**
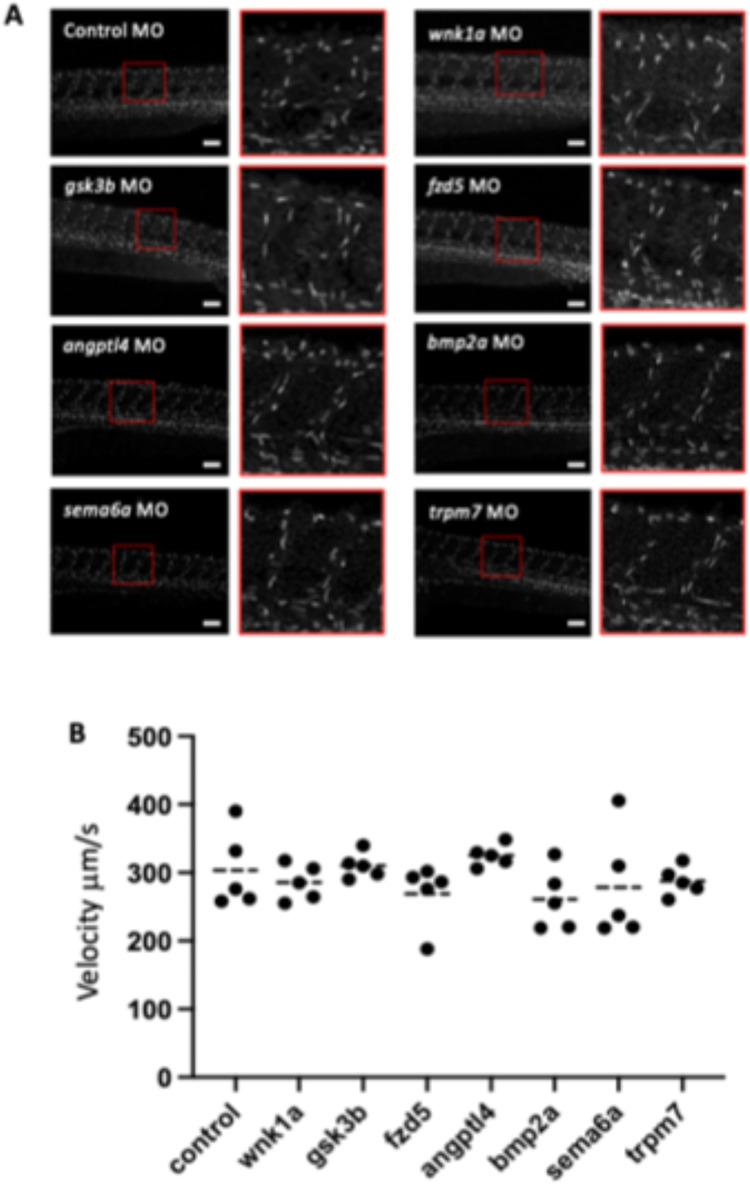
Knock-down of candidate genes does not affect morphology or blood flow in the ISVs. Zebrafish embryos were injected with a gene-specific or non-targeting control MO. (**A**) Morphology of *Tg(fli1a:LifeAct-mClover)* embryos was observed during development and is shown here at 54 hpf. Lateral view, anterior to the left, dorsal up. Scale bar: 100 μm. (**B**) Blood flow was quantified in the ISVs of *Tg(gata1a:dsRed)* morphants at 72 hpf. n = 5 embryos per group.Mean values were compared using a Kruskal–Wallis test, none of the morphants had significantly different blood flow to the control.

Published translation-blocking MOs were identified for *wnk1a, gsk3β, fzd5, sema6a, trpm7* and *angptl4* (Supplemental Table 2) and used to transiently knock down the expression of candidate genes to analyse their potential role in EC proliferation in flow or no flow conditions. It was important to ensure that gene-targeting MOs did not influence EC proliferation indirectly by causing a gross morphological change or by altering flow per se*.* A previous study found that *igf1-*targeting MOs caused gross abnormalities^12^, hence *igf1* was excluded from further analysis. We used a concentration of *wnk1a* MO that did not cause gross morphological changes (Fig. 3A) and did not alter flow in the ISVs of *Tg(gata1:dsRed)* zebrafish that have fluorescently labelled erythrocytes (Supplementary Video 3; Fig. 3B). Similarly, we used concentrations of MOs targeting *gsk3β, fzd5, sema6a, trpm7,* and *angptl4* that did not cause gross morphological changes and did not alter flow in the ISVs (Fig. 3A,B).

**Fig. 4:**
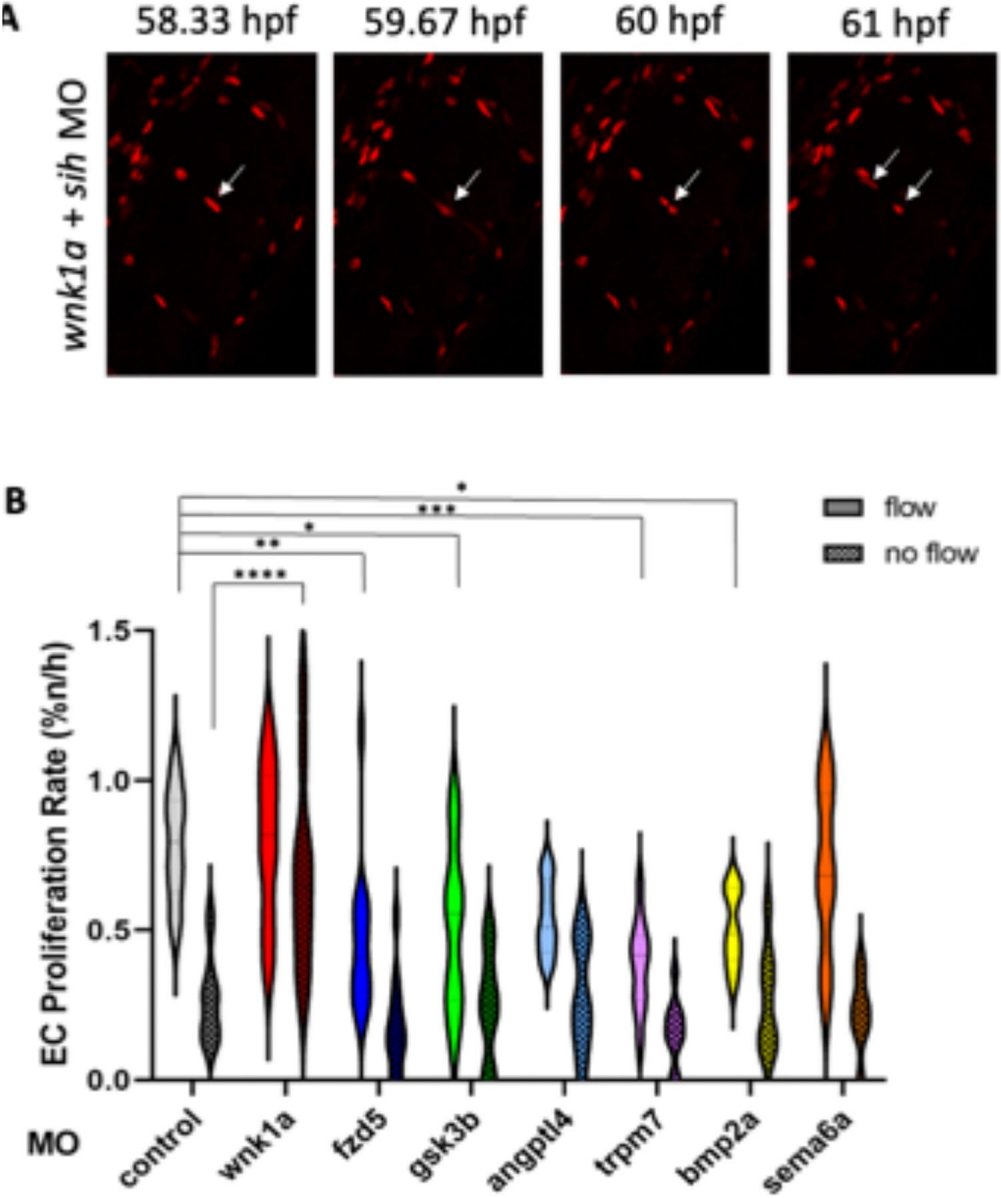
Knock-down of candidate genes influenced EC proliferation in ISVs with and without blood flow. Embryos expressing *Tg(fli1a:nls-mCherry)* with a gene-specific or non-targeting control MO. Some embryos were co-injected with *tnnt2a-*MO to prevent blood flow (no flow), whereas controls did not receive *tnnt2a-*MO (flow). (**A**) Representative images showing a dividing EC (arrows) in a *wnk1a* MO-treated embryo under no flow conditions. (**B**) Proliferation was quantified from 54 to 70 hpf in a minimum of eight embryos per condition. The rate of EC proliferation in the ISVs was quantified as a percentage of nuclei dividing per hour. Data are presented as violin plots. Differences between means were analysed by two-way ANOVA. For the no-flow condition, only significant differences are shown, with non-significant differences omitted. In the flow condition, no statistically significant differences were detected.

Functional screening was performed by MO-targeting of candidate genes in *Tg(fli1a:nls-mCherry)* zebrafish, prior to quantification of EC proliferation under flow or no-flow conditions. MOs targeting *angptl4* and *sema6a* did not significantly impact the rate of EC proliferation under both flow and no flow conditions. However, a potential limitation of this study lies in the relatively low doses of MOs used to target *angptl4* and *sema6a* (to prevent gross morphological changes), which may result in only modest knockdown effects. Consequently, it is not possible to definitively conclude that *angptl4* and *sema6a* are uninvolved in EC proliferation, despite the absence of supporting evidence. Conversely, targeting several other genes produced phenotypes indicative of a regulatory role in EC proliferation. It was observed that knockdown of *wnk1a* in embryos exposed to no-flow led to an increase in proliferation that was similar to levels observed under flow (Fig. 4; Supplementary Videos 4 and 5). By contrast, no significant changes in EC proliferation were observed in response to *wnk1a* knockdown under flow conditions (Fig. 4). Knockdown of *fzd5*, *gsk3β*, *trpm7*, and *bmp2a* led to reduced EC proliferation under flow (Fig. 4). Taken together, these results suggest that *wnk1a* reduces proliferation in no-flow conditions, and *fzd5*, *gsk3β*, *trpm7*, and *bmp2a* promote proliferation in EC exposed to flow.

### WNK1 is enriched at sites of low WSS and in endothelium overlying atherosclerotic plaques

**Fig. 5:**
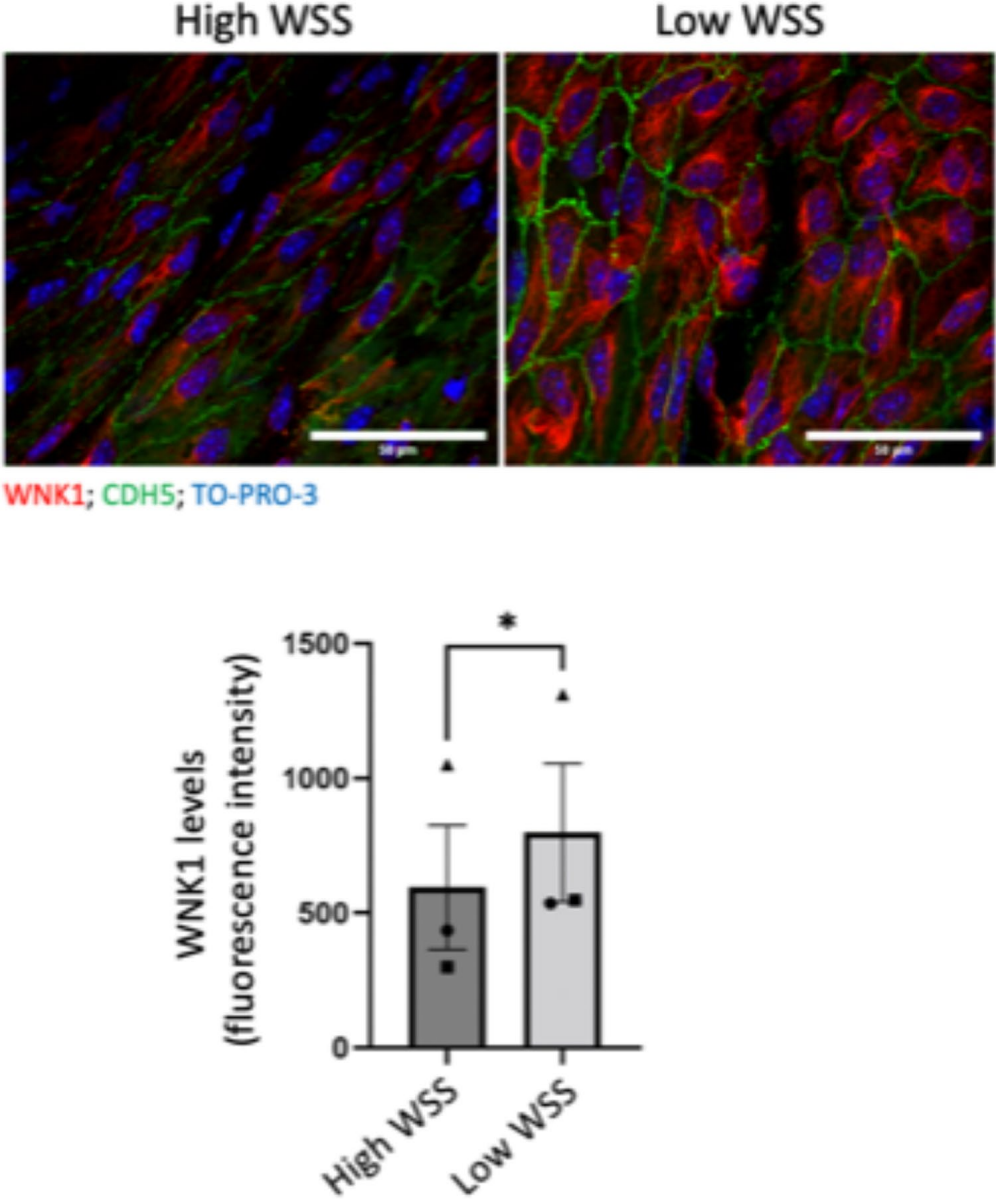
WNK1 is enriched at a low WSS region of the mouse endothelium. WNK1 was analysed by en face staining of the murine aorta using anti-WNK1 antibodies and Alexafluor568-conjugated secondary antibodies (red) (n = 3 mice). EC were co-stained using anti-CDH5 (green) and nuclei were detected with TO-PRO-3 (blue). WNK1 expression was quantified in high and low WSS regions. Mean values + /− standard error of mean are shown. Differences between means were analysed using a paired t-test.

**Fig. 6:**
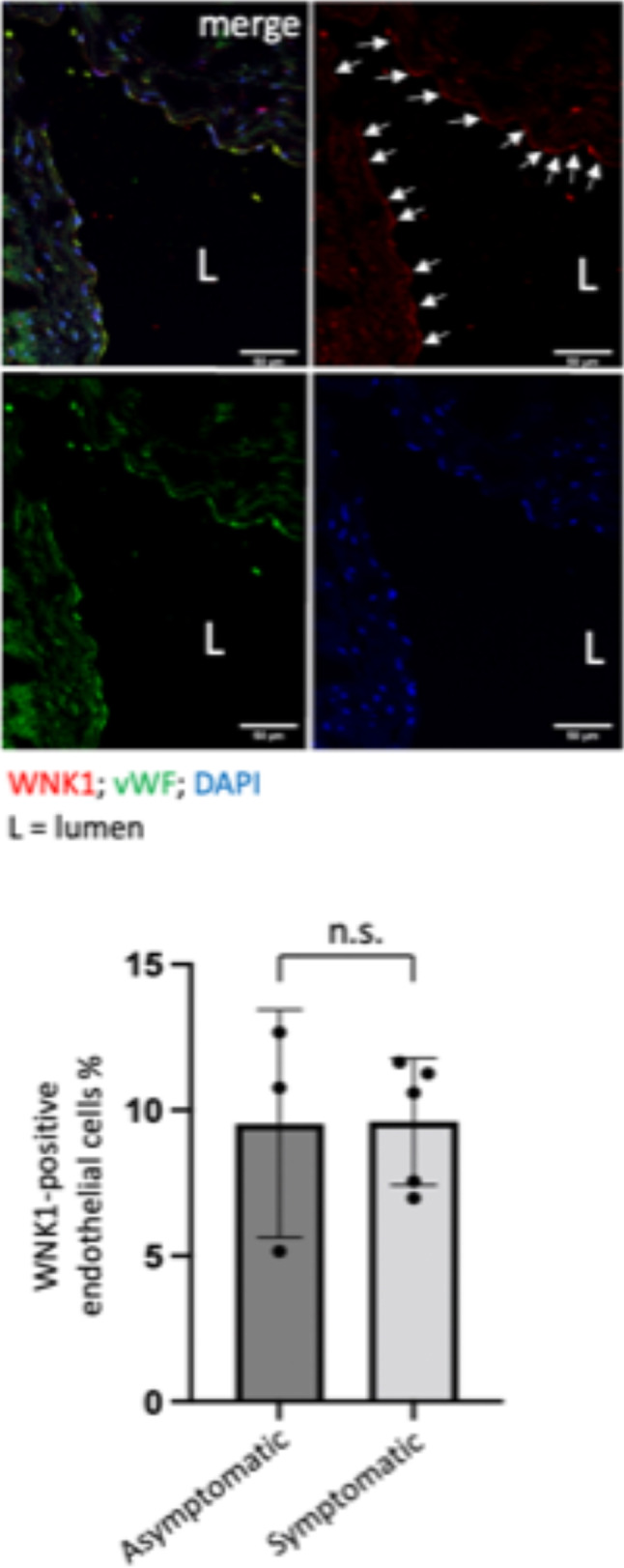
WNK1 was expressed in the endothelium of human atherosclerotic plaques. Endothelial WNK1 expression was determined by immunofluorescent staining of sections of human carotid artery plaques (symptomatic (N = 5); asymptomatic (N = 3) with anti-WNK1 (red) and anti-VWF antibodies (green) to identify EC. Nuclei were counterstained using DAPI (blue). Staining was analysed by fluorescence microscopy and the proportion of WNK1-positive endothelial cells calculated.Representative images are shown. WNK1—positive EC are indicated with arrows. Differences between means were calculated using an unpaired t-test.

*En face* staining was carried out to determine whether WNK1 expression correlates with local haemodynamics of the murine aorta. WNK1 was expressed at higher levels in the low WSS (inner curvature) compared to the high WSS (outer curvature) region (Fig. 5). Furthermore, WNK1 expression in atherosclerotic plaques of human carotid arteries was examined using immunofluorescent staining. Positive staining was observed in various cell types, including ECs overlying the plaque, identified through vWF staining (Fig. 6). Quantitation of endothelial WNK1 expression revealed expression in both asymptomatic and symptomatic atherosclerotic plaques. In summary, these findings demonstrate the expression of WNK1 in ECs overlying atherosclerotic plaques.

## Discussion

Zebrafish embryos have been used to analyse the effect of blood flow on EC apoptosis^12^, EC abundance^14^, EC migration and extrusion^22,23^, however, the mechanisms which transduce flow to regulate zebrafish EC proliferation are poorly understood. The relationship between WSS and EC proliferation has been analysed previously using mammalian cells exposed to flow using in vitro systems. These studies revealed that EC proliferation was reduced by physiological WSS levels (e.g. 1–3 Pa in arterial EC^24,25^), but enhanced by supraphysiological mechanical force (e.g. 1.6 Pa in venous EC^26^; e.g. 28 Pa in arterial EC^27^). Here we analysed EC proliferation under flow and no-flow conditions in zebrafish ISV using a real-time microscopy method that we previously optimised^15^. An intrinsic limitation of the live zebrafish embryo imaging system is the potential influence of embryo embedding on EC proliferation. Nevertheless, we found this system useful for analyzing proliferation under modified flow conditions and for identifying genes implicated in these processes. By analysing ISVs exposed to flow versus no-flow conditions, we demonstrate that the rate of EC proliferation was significantly reduced in the absence of flow. Previous studies from our group revealed that the cessation of blood flow leads to fewer EC in zebrafish ISVs^14,28^, however the mechanism was not established. Serbanovic-Canic et al.^12^ indicate that elevated apoptosis could be partially responsible for the reduced number of EC under flow cessation, and here we demonstrate that flow cessation also reduces EC proliferation. Thus reduced EC numbers in static conditions is likely due to the combination of reduced proliferation and increased apoptosis.

WSS modifies proliferation by altering multiple signalling pathways including p53^24,25^, AMPK^29^ and YAP/TAZ^30^. Here we coupled the zebrafish ISV flow model to MO-based screening to analyse the potential function role of several flow-regulated genes. These genes were selected on the basis that they were upregulated in a low WSS region of the porcine aorta and were expressed in zebrafish EC. A limitation of the study is that qRT-PCR analysis of sorted zebrafish embryo EC gave variable results with some genes exhibiting EC expression in only 1 of 3 experiments. This is likely due to low levels of gene expression that were on the borderline of our qRT-PCR test. Since the purpose of the study was to screen for gene function we chose to incorporate all genes that exhibited EC expression into subsequent functional analysis.

**Fig. 7:**
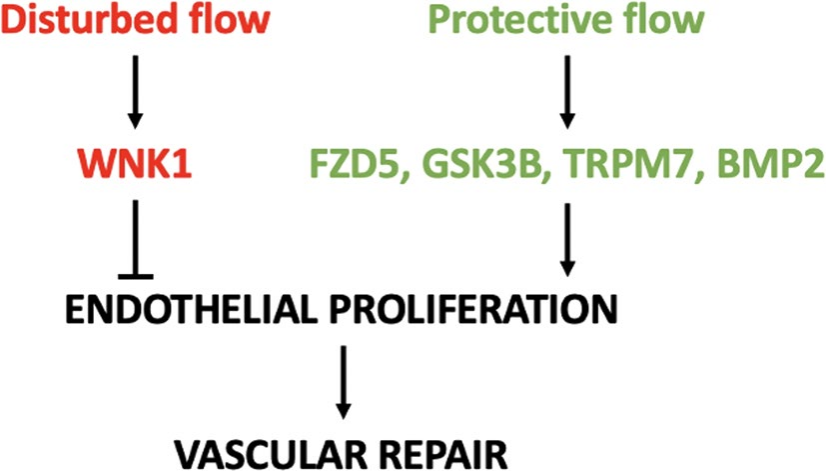
Schematic summary.

Knockdown of *fzd5, gsk3b, trpm7*, and *bmp2* resulted in reduced EC proliferation, specifically in ISVs subjected to flow. Conversely, knockdown of *wnk1a* in embryos led to increased EC proliferation, specifically in ISVs under no-flow conditions. Therefore, *fzd5, gsk3b, trpm7*, and *bmp2* positively regulate EC proliferation in the presence of flow, while *wnk1a* negatively regulates EC proliferation in the absence of blood flow. These findings have implications for vascular repair, which is dependent on EC proliferation (Fig. 7); however, further investigation is necessary to directly elucidate their roles in the vascular repair process. Studies using MOs should be interpreted with caution since they can have off-target effects by upregulating the cell cycle inhibitor p53^31^. It is notable, however, that the *wnk1a* MO led to increased proliferation which is not consistent with activation of p53. Additionally, the effects of MO injection were compared to a non-targeting control MO, suggesting that the effect of the *wnk1a* MO may accurately reflect wnk1a biology. However, additional research is necessary, including the analysis of *wnk1* overexpression driven by an endothelial-specific promoter, to definitively confirm the role of *wnk1* in EC proliferation.

Our observation that *wnk1* is expressed in endothelium of zebrafish embryos and controls EC proliferation in ISVs is consistent with previous research demonstrating that *wnk1* knockdown causes aberrant angiogenesis in several vascular beds including ISVs.^5^ However, the precise role of WNK1 in angiogenesis including its role in tip and stalk cell biology is uncertain. WNK1 knockdown also reduces angiogenesis in human hepatoma cells xenotransplanted into zebrafish embryos^32^. Consistently, we observed altered vascular structures in response to relatively higher doses of *wnk1* MO, whereas the lower doses that we used for functional screening did not alter vascular morphology. Our finding that *wnk1a* reduces EC proliferation in zebrafish embryos under conditions of flow cessation is consistent with previous observations that WNK1 negatively regulates proliferation of endometrial cells and adipocytes^33,34^. However, WNK1 can promote the proliferation in venous EC^8^ and other cell types^35,36^, suggesting that the control of proliferation by WNK1 is dependent on cellular and physiological context. Thus, although *wnk1* enhances EC proliferation in angiogenesis, we observed that it reduces proliferation in embryonic vasculature that lacks flow. We speculate that this ensures that vessel growth and maturation is restricted to those vessels that have developed correctly and are perfused, and that malformed vessels without perfusion are not selected for further growth. Recent research demonstrated that WNK kinases can be activated by hydrostatic pressure which alters the dimer to monomer equilibrium^37^. It is therefore plausible that WNK1a may be directly activated by flow-induced mechanical force in zebrafish, or alternatively that it is activated downstream from other mechanosensors^38^. An outstanding question is whether WNK1 exhibits altered expression during vascular injury to control EC repair processes and this should be analysed in subsequent research. WNK1 is regulated by VEGF-AKT signalling during angiogenesis^2,5^ and it would be interesting to know if these factors also control WNK1 during vascular repair.

We observed that WNK1 is enriched in disease-prone regions of adult murine arteries and is also expressed in the endothelium of human carotid artery plaques. These findings suggest that WNK1 may play a role in EC function at disease-prone sites and within plaques, potentially by regulating EC proliferation. There is considerable interest in the development of WNK1 inhibitors to treat cancer and hypertension^39^, and it will be interesting in future studies to assess whether WNK1 inhibitors can reduce atherosclerosis progression.

## Electronic supplementary material

Below is the link to the electronic supplementary material.

Supplementary Material 1

Supplementary Material 2

Supplementary Material 3

Supplementary Material 4

Supplementary Material 5

Supplementary Material 6

## Data Availability

All data are included within the manuscript or supplementary information files.
