# Supplementary material for: Zebrafish model for functional screening of flow-responsive genes controlling endothelial cell proliferation
Source: Sci Rep. 2024 Dec 3;14:30130. doi: 10.1038/s41598-024-77370-1 (PMC11615307; doi:10.1038/s41598-024-77370-1)
Supplement: Supplementary file 1 — Supplementary Material 1 [file 41598_2024_77370_MOESM1_ESM.docx]

**SUPPLEMENTAL MATERIALS**

**LEGENDS FOR SUPPLEMENTARY VIDEOS**

**Supplementary Video 1: EC proliferation timelapse in zebrafish intersegmental vessels with flow.** EC proliferation was quantified in endothelial cells of Tg(fli1a:LifeAct-mClover; fli1a:nls-mCherry) embryos treated with control morpholino. EC proliferation was imaged from 54 to 70 hpf, proliferation was quantified by determining the number of nuclei division events as a percentage of total ISV nuclei.

**Supplementary Video 2: EC proliferation timelapse in zebrafish intersegmental vessels with no-flow.** EC proliferation was quantified in endothelial cells of *Tg(fli1a:LifeAct-mClover; fli1a:nls-mCherry)* embryos treated with *tnnt2a* morpholino. EC proliferation was imaged from 54 to 70 hpf, proliferation was quantified by determining the number of nuclei division events as a percentage of total ISV nuclei.

**Supplementary Video 3: Intersegmental blood flow measurement by tracking erythrocytes.** Blood flow was quantified in the ISVs by imaging the ISVs of *Tg(gata1a:dsRed)* embryos at high speed and tracking erythrocytes using TrackMate. Blood flow quantification was done at 72 hpf on embryos treated with MOs targeting each gene of interest.

**Supplementary Video 4: EC proliferation timelapse in zebrafish intersegmental vessels with flow and wnk1a MO.**

EC proliferation was quantified in endothelial cells of Tg(fli1a:LifeAct-mClover; fli1a:nls-mCherry) embryos treated with *wnk1a* morpholino (MO). EC proliferation was imaged from 54 to 70 hpf, proliferation was quantified by determining the number of nuclei division events as a percentage of total ISV nuclei.

**Supplementary Video 5: EC proliferation timelapse in zebrafish intersegmental vessels with no-flow and wnk1a MO.**

EC proliferation was quantified in endothelial cells of Tg(fli1a:LifeAct-mClover; fli1a:nls-mCherry) embryos treated with *tnnt2a* and *wnk1a* morpholinos (MO). EC proliferation was imaged from 54 to 70 hpf, proliferation was quantified by determining the number of nuclei division events as a percentage of total ISV nuclei.

**Supplemental Table 1: qRT-PCR primer sequences**

| **Gene of interest** | **Morpholino sequence (5’-3’)** | **Dose (ng)** | **Publication** |
| --- | --- | --- | --- |
| *tnnt2a* | CATGTTTGCTCTGATCTGACACGCA | 1.0 | ^1^ |
| non targeting control | CCTCTTACCTCAGTTATTTATA | 1.75 | ^1^ |
| *wnk1a* | ACTTGACCATCTTGTCGTTGAGATT | 1.75 | ^2^ |
| *gsk3b* | GTTCTGGGCCGACCGGACATTTTTC | 1.75 | ^3^ |
| *fzd5* | GATGCTCGTCTGCAGGTTTCCTCAT | 3.5 | ^4^ |
| *sema6a* | TGCTGATATCCTGCACTCACCTCAC | 2.0 | ^5^ |
| *trpm7* | ATCCAGGACTTCTGGGACATTCT | 1.75 | ^6^ |
| *bmp2a* | TGGACGAGACCATGATGATCTCTGC | 3.125 | ^7^ |
| *angptl4* | TCAGCAATGATAAACTGACTTACCA | 2.5 | ^1^ |

**Supplemental Table 2: Morpholino sequences**

**Supplemental Table 3: Proliferation linked genes enriched at a low WSS region and their zebrafish orthologues**

**REFERENCES FOR SUPPLEMENTAL MATERIALS**

1. Serbanovic-Canic J, de Luca A, Warboys C, Ferreira PF, Luong LA, Hsiao S, Gauci I, Mahmoud M, Feng S, Souilhol C, Bowden N, Ashton JP, Walczak H, Firmin D, Krams R, Mason JC, Haskard DO, Sherwin S, Ridger V, Chico TJ, Evans PC. Zebrafish Model for Functional Screening of Flow-Responsive Genes. *Arterioscler Thromb Vasc Biol* 2017;**37**:130-143.

2. Lai JG, Tsai SM, Tu HC, Chen WC, Kou FJ, Lu JW, Wang HD, Huang CL, Yuh CH. Zebrafish WNK lysine deficient protein kinase 1 (wnk1) affects angiogenesis associated with VEGF signaling. *PLoS One* 2014;**9**:e106129.

3. Lee HC, Tsai JN, Liao PY, Tsai WY, Lin KY, Chuang CC, Sun CK, Chang WC, Tsai HJ. Glycogen synthase kinase 3 alpha and 3 beta have distinct functions during cardiogenesis of zebrafish embryo. *BMC Dev Biol* 2007;**7**:93.

4. Cavodeassi F, Carreira-Barbosa F, Young RM, Concha ML, Allende ML, Houart C, Tada M, Wilson SW. Early stages of zebrafish eye formation require the coordinated activity of Wnt11, Fz5, and the Wnt/beta-catenin pathway. *Neuron* 2005;**47**:43-56.

5. Ebert AM, Childs SJ, Hehr CL, Cechmanek PB, McFarlane S. Sema6a and Plxna2 mediate spatially regulated repulsion within the developing eye to promote eye vesicle cohesion. *Development* 2014;**141**:2473-2482.

6. Sah R, Mesirca P, Van den Boogert M, Rosen J, Mably J, Mangoni ME, Clapham DE. Ion channel-kinase TRPM7 is required for maintaining cardiac automaticity. *Proc Natl Acad Sci U S A* 2013;**110**:E3037-3046.

7. Quillien A, Blanco-Sanchez B, Halluin C, Moore JC, Lawson ND, Blader P, Cau E. BMP signaling orchestrates photoreceptor specification in the zebrafish pineal gland in collaboration with Notch. *Development* 2011;**138**:2293-2302.
