# Supplementary figures and images for: Zebrafish model for functional screening of flow-responsive genes controlling endothelial cell proliferation
Source: Sci Rep. 2024 Dec 3;14:30130. doi: 10.1038/s41598-024-77370-1 (PMC11615307; doi:10.1038/s41598-024-77370-1)

## Slide 1
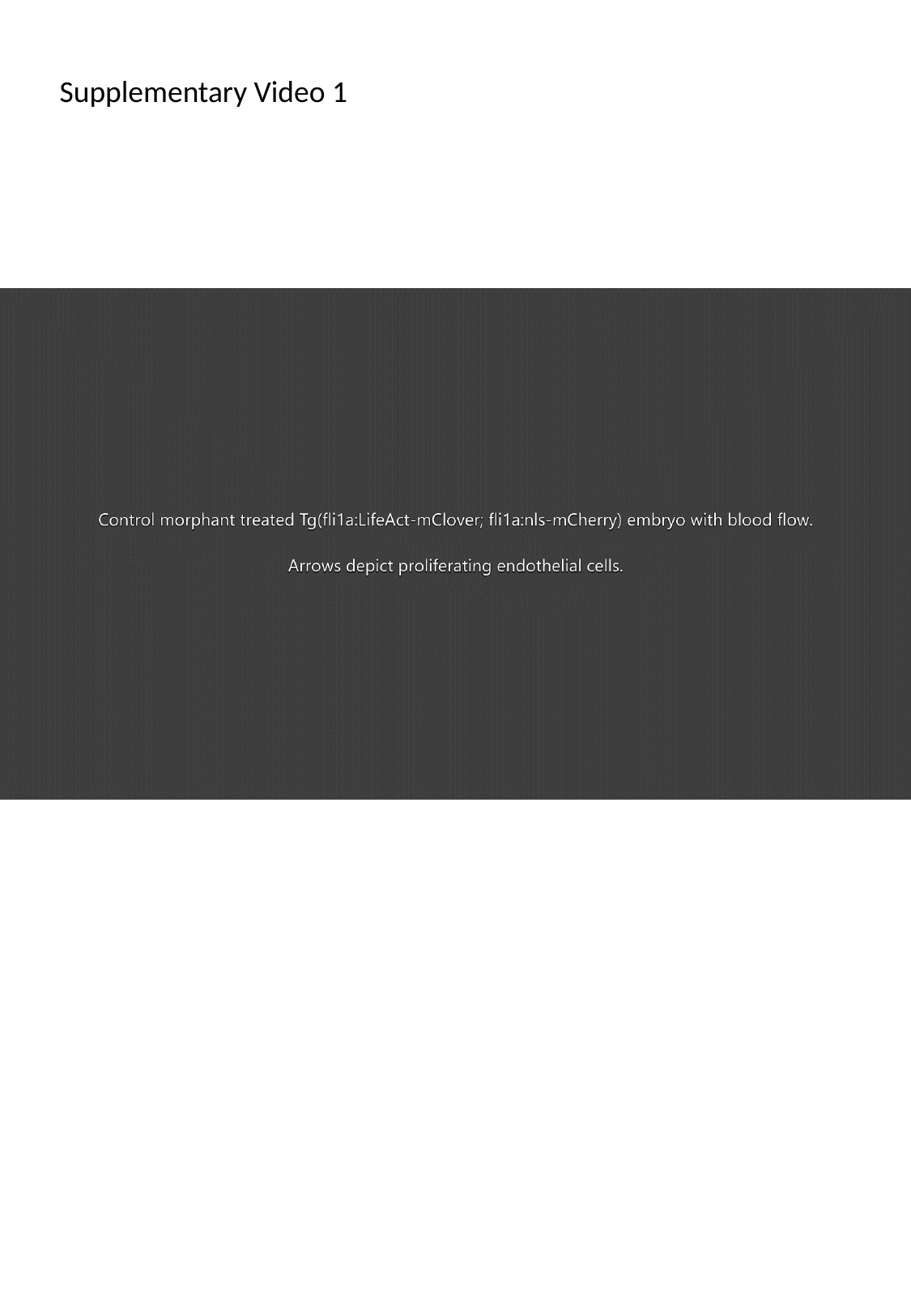

Supplementary Video 1

Supplement: Supplementary file 2 — Supplementary Material 2 [file 41598_2024_77370_MOESM2_ESM.pptx]

## Slide 1
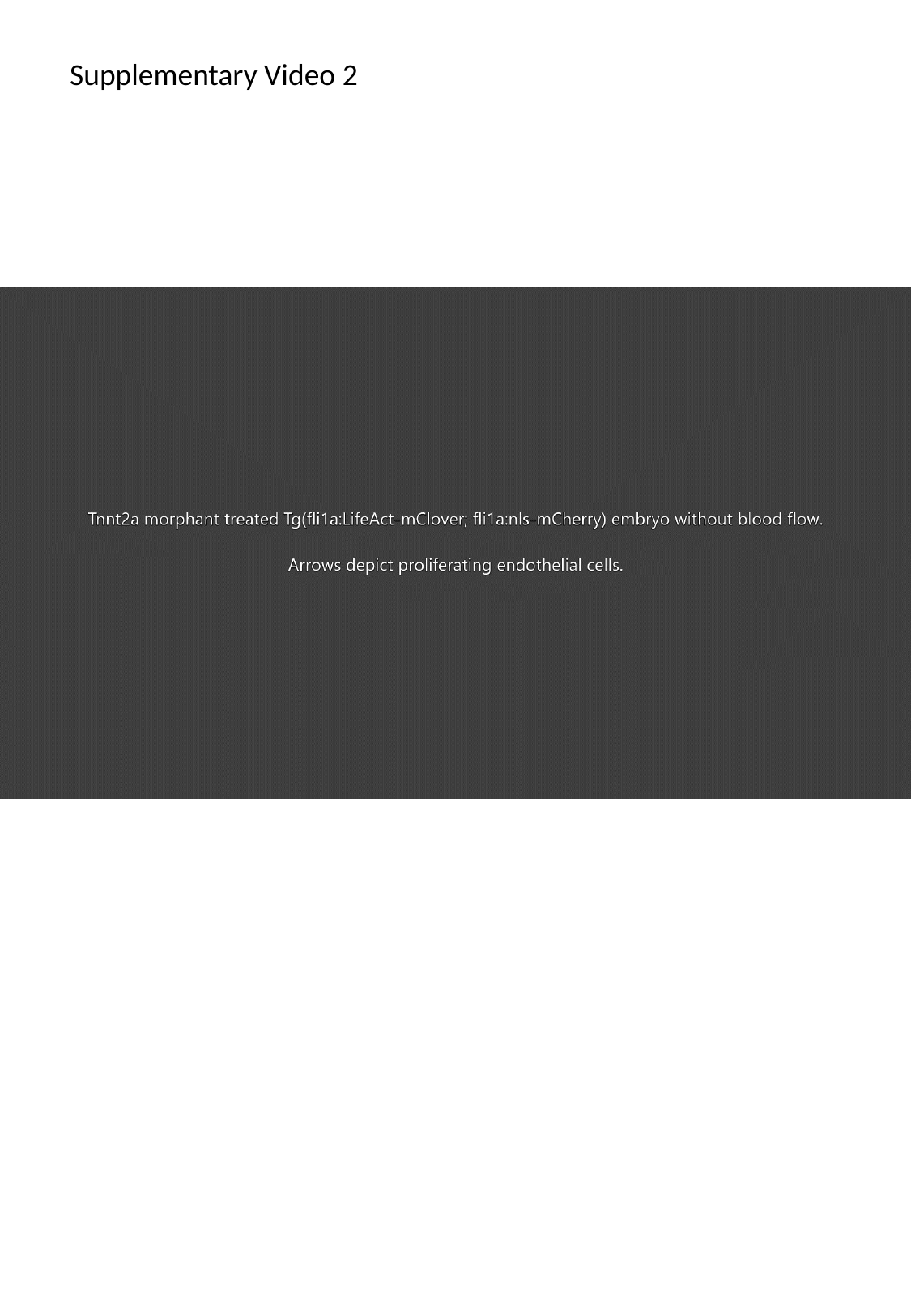

Supplementary Video 2

Supplement: Supplementary file 3 — Supplementary Material 3 [file 41598_2024_77370_MOESM3_ESM.pptx]
